# Supplementary material for: Effect of a Multicomponent Intervention with Tele-Rehabilitation and the Vivifrail© Exercise Programme on Functional Capacity after Hip Fracture: Study Protocol for the ActiveFLS Randomized Controlled Trial
Source: J Clin Med. 2023 Dec 23;13(1):97. doi: 10.3390/jcm13010097 (PMC10779784; doi:10.3390/jcm13010097)
Supplement: Supplementary file 1 [file jcm-13-00097-s001.zip › jcm-2672023-supplementary.pdf]

Supplementary Material

Supplementary Material S1: Spirit Checklist

SPIRIT 2013 Checklist: Recommended items to address in a clinical trial protocol and related documents*

Section/item	Item Nº	Description	Addressed on page number
**Administrative information**			
Title	1	Descriptive title identifying the study design, population, interventions, and, if applicable, trial acronym	**1**
Trial registration	2a	Trial identifier and registry name. If not yet registered, name of intended registry	**3**
	2b	All items from the World Health Organization Trial Registration Data Set	**3 and Supplementary Material 2**
Protocol version	3	Date and version identifier	**3**
Funding	4	Sources and types of financial, material, and other support	**3 and 13**
Roles and responsibilities	5a	Names, affiliations, and roles of protocol contributors	**3**
	5b	Name and contact information for the trial sponsor	**13**
	5c	Role of study sponsor and funders, if any, in study design; collection, management, analysis, and interpretation of data; writing of the report; and the decision to submit the report for publication, including whether they will have ultimate authority over any of these activities	**13**
	5d	Composition, roles, and responsibilities of the coordinating centre, steering committee, endpoint adjudication committee, data management team, and other individuals or groups overseeing the trial, if applicable (see Item 21a for data monitoring committee)	**10**
**Introduction**			
Background and rationale	6a	Description of research question and justification for undertaking the trial, including summary of relevant studies (published and unpublished) examining benefits and harms for each intervention	**3-4**
	6b	Explanation for choice of comparators	**5-6**
Objectives	7	Specific objectives or hypotheses	**4**

Trial design	8	Description of trial design including type of trial (eg, parallel group, crossover, factorial, single group), allocation ratio, and framework (eg, superiority, equivalence, noninferiority, exploratory)	**4-5**
**Methods: Participants, interventions, and outcomes**			
Study setting	9	Description of study settings (eg, community clinic, academic hospital) and list of countries where data will be collected. Reference to where list of study sites can be obtained	**4**
Eligibility criteria	10	Inclusion and exclusion criteria for participants. If applicable, eligibility criteria for study centres and individuals who will perform the interventions (eg, surgeons, psychotherapists)	**4-5**
Interventions	11a	Interventions for each group with sufficient detail to allow replication, including how and when they will be administered	**5,6**
	11b	Criteria for discontinuing or modifying allocated interventions for a given trial participant (eg, drug dose change in response to harms, participant request, or improving/worsening disease)	**6**
	11c	Strategies to improve adherence to intervention protocols, and any procedures for monitoring adherence (eg, drug tablet return, laboratory tests)	**7**
	11d	Relevant concomitant care and interventions that are permitted or prohibited during the trial	**7**
Outcomes	12	Primary, secondary, and other outcomes, including the specific measurement variable (eg, systolic blood pressure), analysis metric (eg, change from baseline, final value, time to event), method of aggregation (eg, median, proportion), and time point for each outcome. Explanation of the clinical relevance of chosen efficacy and harm outcomes is strongly recommended	**7-9**
Participant timeline	13	Time schedule of enrolment, interventions (including any run-ins and washouts), assessments, and visits for participants. A schematic diagram is highly recommended (see Figure)	**Figure 1**
Sample size	14	Estimated number of participants needed to achieve study objectives and how it was determined, including clinical and statistical assumptions supporting any sample size calculations	**9**

Recruitment	15	Strategies for achieving adequate participant enrolment to reach target sample size	**4-5**

Methods: Assignment of interventions (for controlled trials)

Allocation:			
Sequence generation	16a	Method of generating the allocation sequence (eg, computer-generated random numbers), and list of any factors for stratification. To reduce predictability of a random sequence, details of any planned restriction (eg, blocking) should be provided in a separate document that is unavailable to those who enrol participants or assign interventions	**9**
Allocation concealment mechanism	16b	Mechanism of implementing the allocation sequence (eg, central telephone; sequentially numbered, opaque, sealed envelopes), describing any steps to conceal the sequence until interventions are assigned	**9**
Implementation	16c	Who will generate the allocation sequence, who will enrol participants, and who will assign participants to interventions	**9**
Blinding (masking)	17a	Who will be blinded after assignment to interventions (eg, trial participants, care providers, outcome assessors, data analysts), and how	**9**
	17b	If blinded, circumstances under which unblinding is permissible, and procedure for revealing a participant's allocated intervention during the trial	**9**

Methods: Data collection, management, and analysis

Data collection methods	18a	Plans for assessment and collection of outcome, baseline, and other trial data, including any related processes to promote data quality (eg, duplicate measurements, training of assessors) and a description of study instruments (eg, questionnaires, laboratory tests) along with their reliability and validity, if known. Reference to where data collection forms can be found, if not in the protocol	**9-10**
	18b	Plans to promote participant retention and complete follow-up, including list of any outcome data to be collected for participants who discontinue or deviate from intervention protocols	**9-10**

Data management	19	Plans for data entry, coding, security, and storage, including any related processes to promote data quality (eg, double data entry; range checks for data values). Reference to where details of data management procedures can be found, if not in the protocol	**9-10**
Statistical methods	20a	Statistical methods for analysing primary and secondary outcomes. Reference to where other details of the statistical analysis plan can be found, if not in the protocol	**10**
	20b	Methods for any additional analyses (eg, subgroup and adjusted analyses)	**10**
	20c	Definition of analysis population relating to protocol non-adherence (eg, as randomised analysis), and any statistical methods to handle missing data (eg, multiple imputation)	**10**
**Methods: Monitoring**			
Data monitoring	21a	Composition of data monitoring committee (DMC); summary of its role and reporting structure; statement of whether it is independent from the sponsor and competing interests; and reference to where further details about its charter can be found, if not in the protocol. Alternatively, an explanation of why a DMC is not needed	**10**
	21b	Description of any interim analyses and stopping guidelines, including who will have access to these interim results and make the final decision to terminate the trial	**10**
Harms	22	Plans for collecting, assessing, reporting, and managing solicited and spontaneously reported adverse events and other unintended effects of trial interventions or trial conduct	**11**
Auditing	23	Frequency and procedures for auditing trial conduct, if any, and whether the process will be independent from investigators and the sponsor	**11**
**Ethics and dissemination**			
Research ethics approval	24	Plans for seeking research ethics committee/institutional review board (REC/IRB) approval	**12**
Protocol amendments	25	Plans for communicating important protocol modifications (eg, changes to eligibility criteria, outcomes, analyses) to relevant parties (eg, investigators, REC/IRBs, trial participants, trial registries, journals, regulators)	**25**

Consent or assent	26a	Who will obtain informed consent or assent from potential trial participants or authorised surrogates, and how (see Item 32)	**5**
	26b	Additional consent provisions for collection and use of participant data and biological specimens in ancillary studies, if applicable	**5**
Confidentiality	27	How personal information about potential and enrolled participants will be collected, shared, and maintained in order to protect confidentiality before, during, and after the trial	**10**
Declaration of interests	28	Financial and other competing interests for principal investigators for the overall trial and each study site	**12**
Access to data	29	Statement of who will have access to the final trial dataset, and disclosure of contractual agreements that limit such access for investigators	**12**
Ancillary and post-trial care	30	Provisions, if any, for ancillary and post-trial care, and for compensation to those who suffer harm from trial participation	**7**
Dissemination policy	31a	Plans for investigators and sponsor to communicate trial results to participants, healthcare professionals, the public, and other relevant groups (eg, via publication, reporting in results databases, or other data sharing arrangements), including any publication restrictions	**11**
	31b	Authorship eligibility guidelines and any intended use of professional writers	**11**
	31c	Plans, if any, for granting public access to the full protocol, participant-level dataset, and statistical code	**11**
**Appendices**			
Informed consent materials	32	Model consent form and other related documentation given to participants and authorized surrogates	**Supplementary Material 3**
Biological specimens	33	Plans for collection, laboratory evaluation, and storage of biological specimens for genetic or molecular analysis in the current trial and for future use in ancillary studies, if applicable	**8**

Supplementary Material S2: Items from the World Health Organization Trial Registration Dataset

Data category	Information
Primary registry and trial identifying number	ClinicalTrials.gov NCT05435534
Date of registration in primary registry	25 May, 2022
Secondary identifying numbers	--
Source(s) of monetary or material support	"La Caixa" Foundation (ID 100010434)
Primary sponsor	"La Caixa" Foundation
Secondary sponsor(s)	--
Contact for public queries	Bernardo Abel Cedeño Veloz, MD email: ba.cedeno.veloz@navarra.es
Contact for scientific queries	Bernardo Abel Cedeño Veloz, MD, Geriatric Unit, Hospital Universitario de Navarra, Pamplona (Spain)
Public title	Effect of a multicomponent intervention with telerehabilitation and Vivifrail on functional capacity after hip fracture: study protocol for ActiveFLS randomized control trial
Scientific title	multicomponent intervention in patients with hip fracture -randomized controlled trial
Countries of recruitment	Spain
Health condition(s) or problem(s) studied	Hip fracture, frailty
Intervention(s)	*Control group*: normal hospital care<br>*Intervention group*: multicomponent intervention with home-based exercise
Key inclusion and exclusion criteria	The inclusion criteria are:<br>- Age: 75 years or older with diagnosis of fragility hip fracture<br>- Barthel Index for Activities of Daily Living (ADLs) score of 60 or more - Independence to mobility using the FAC of 3 or more<br>- Ability/Support to use Active HIP app.<br>- Informed consent by patients, relatives, or legal representatives<br><br>The exclusion criteria are:<br>- Moderate-severe cognitive impairment considered as a Goldberg Global Deterioration Scale score ≥ 5.<br>- Secondary osteoporosis.<br>- Institutionalized in permanent nursing home.<br>- Refusal to sign informed consent by patient/primary caregiver/legal guardian or inability to obtain it.

	- Terminal illness (life expectancy less than 3 months)<br>- Any factor precluding performance of physical exercise. These factors include: Acute myocardial infarction in the past three months or unstable angina, severe heart valve insufficiency, arrhythmia or uncontrolled arterial hypertension, pulmonary embolism in the past 3 months, hemodynamic instability
Study type	-Interventional<br>-Allocation: randomized, Intervention model: parallel assignment; Masking: double blinded<br>-Primary purpose: treatment<br>-Phase III
Date of first enrolment	June, 2022
Target sample size	174
Recruitment status	Recruiting
Primary outcome(s)	-Change in functional status during the study period. The functional capacity of patients will be evaluated by the Short Physical Performance Battery (timeframe:12 months)
Key secondary outcomes	- Functional status (timeframe:12 months)<br>- Cognitive status (timeframe:12 months)<br>- Mood status (timeframe:12 months)<br>-Mortality (timeframe:12 months)<br>-Quality of life (timeframe:12 months)<br>-Use of health sources (timeframe:12 months)<br>-Falls (timeframe:12 months)

Administrative information

Title {1}	Effect of a multicomponent intervention with tele-rehabilitation and the Vivifrail exercise programme on functional capacity after hip fracture: Study protocol for the ActiveFLS randomized controlled trial.
Trial registration {2a and 2b}	NCT05435534 (Date of registration 25.05.2022).
Protocol version {3}	20 February 2023. Version 2.
Funding {4}	This research did not receive any specific grant from funding agencies in the public or not-for-profit sectors.
Author details {5a}	1 Navarre University Hospital (HUN), Irunlarrea 3, 31008 Pamplona, Navarra, Spain.<br>2 Navarrabiomed, Institute for Health Research of Navarra (IDISNA), Irunlarrea 3, 31008 Pamplona, Navarra, Spain.

	3 Public University of Navarre, Av Cataluña s/n 31006 Pamplona, Navarra, Spain.<br><br>4 Department of Orthopaedics Clinics and Traumatology, University Hospital of Navarre (HUN), Pamplona, Navarra, Spain.<br><br>5 CIBER of Frailty and Healthy Aging (CIBERFES), Instituto de Salud Carlos III, Av Monforte de Lemos, 3-5, Pabellón 11, Planta 0, 28029 Madrid, Spain.<br><br>6 Institute for Health Research of Navarre (IDISNA), Irunlarrea 3, 31008 Pamplona, Navarra, Spain.

Supplementary Material S3: Informed Consent

Effect of a multicomponent intervention with tele-rehabilitation and the Vivifrail© exercise programme on functional capacity after hip fracture: Study protocol for the ActiveFLS randomized controlled trial

Informed Consent form for patients and caregivers

This Informed Consent Form is for men and women who attend Hospital Universitario de Navarra (HUN) in Pamplona (Spain) or their caregivers, and who we are inviting to participate in research on delirium. The title of our research project is "Effect of a multicomponent intervention with telerehabilitation and Vivifrail on functional capacity after hip fracture: study protocol for ActiveFLS randomized control trial".

Principal Investigator: Bernardo Abel Cedeño Veloz

Organization: Navarrabiomed, Fundación Miguel Servet

Sponsor: Fundación "La Caixa"

Version 2

This Informed Consent Form has two parts:

I. Information Sheet (to share information about the research with you)
II. Certificate of Consent (for signatures if you agree to take part)

You will be given a copy of the full Informed Consent Form

PART I: INFORMATION SHEET

Introduction

I am Bernardo Abel Cedeño Veloz, a geriatrician working for Navarrabiomed Research Institute. We are doing research on hip fracture, which is a very common disease in this country. I am going to give you information and invite you to be part of this research. Before you decide, you can talk to anyone you feel comfortable with about the research.

There may be some words that you do not understand. Please ask me to stop as we go through the information and I will take time to explain. If you have questions later, you can ask them of me or the staff.

Purpose of the research

Hip fractures is the kind of fracture more related to hospitalization, high costs, mortality, and functional decline. Many guidelines for prevention of new fractures and functional recovery exist nowadays; with special focus on osteoporosis treatment (such as fracture liaison services). However, most health

systems were too fragmented and unable to guarantee the adequate management of frail and complex individuals at risk of or suffering from fragility fractures

The reason we are doing this research is to find out if a multicomponent program based on home-based exercise with telerehabilation is better than usual care which is currently being used, during hip fracture follow-up.

Type of Research Intervention

This research will involve a questionnaire, five blood tests, two densitometry scans and a physical exercise program at home.

Participant selection

We are inviting all older adults with hip fracture able to walk and with good grade of independent who attend Hospital Universitario de Navarra (HUN) to participate in this study.

Voluntary Participation

Your participation in this research is entirely voluntary. It is your choice whether to participate or not. Whether you choose to participate or not, all the services you receive at this hospital will continue and nothing will change. If you choose not to participate in this research project, you will be offered the treatment that is routinely offered in this hospital for hip fracture. You may change your mind later and stop participating even if you agreed earlier.

Procedures and Protocol

Because we do not know the best pathway for functional recovery after hip fracture, we need to compare both alternatives. To do this, we will put people taking part in this research into two groups. The groups are selected by chance, as if by tossing a coin.

In the active control care arm (control), participants will receive outpatient care according to usual clinical practice. In the intervention arm (ActiveFLS), participants will receive, besides usual care, a multicomponent physical exercise program based on the ActiveHip+ for 3 months. In subsequent revisions, after finishing ActiveHip+ program, Vivifrail program will be given according to the patient's functional capacity. There will also be a protocolized nutritional assessment and intervention, adjustment of polypharmacy according to STOP/START criteria, screening for anxiety/depression, fear of falling and cognitive deterioration, as well as protocolized secondary fracture prevention treatment. All subjects are permitted to continue their usual physical activity during the study.

It is important that neither you nor we know which treatment you receive. This information will be in our files, but we will not look at these files until after the research is finished. This is the best way we have for testing without being influenced by what we think or hope might happen. We will then compare which of the two has the best results. The healthcare workers will be looking after you and the other participants very carefully during the study. If there is anything you are concerned about or that is bothering you about the research please talk to me or one of the other researchers.

Description of the Process: If you wish to participate, your medical history will be consulted, and several questionnaires will be carried out. The study will have four major data collection points (baseline during acute hospitalization, 3, 6, and 12 months) and 1 minor point (1 month). Likewise, a blood test will be taken on admission and in ever major data collection (at the end of the research, in 1 year, any left-over blood sample will be destroyed). Also, a densitometry scan will be done at minor point and 12 months.

Duration

The research takes place during 1 year after discharge in total. During that time, it will be necessary to do some questionnaires for follow-up by out clinic (1, 3, 6, and 12 months after discharge).

Side Effects

Side effects during/after this exercise programme are very unusual muscle (pain, fatigue and general aches). However, we will follow you closely and keep track of any unwanted effects or any problems.

Risks

By participating in this research, it is possible that you will be at greater risk of having pain, fatigue or general aches. While the possibility of this happening is very low, you should still be aware of the possibility. We will try to decrease the chances of this event occurring, but if something unexpected happens, we will stop physical training and provide you whatever you need.

Benefits

If you participate in this research, you will benefit from the advantages of multicomponent intervention with physical exercise: you will improve your functional status, strength, and other health parameters. There may not be any benefit to older adults with hip fracture at this stage of the research, but future generations are likely to benefit.

Reimbursements

You will not be given any money or gifts to take part in this research.

Confidentiality

The information that we collect from this research project will be kept confidential. Information about you that will be collected during the research will be put away and no-one but the researchers will be able to see it. Any information about you will have a number on it instead of your name. Only the researchers will know what your number is and we will lock that information up with a lock and key.

Sharing the Results

The knowledge that we get from doing this research will be shared with you through community meetings before it is made widely available to the public. Confidential information will not be shared.

There will be small meetings in the community and these will be announced. After these meetings, we will publish the results in order that other interested people may learn from our research.

Right to Refuse or Withdraw

You do not have to take part in this research if you do not wish to do so. You may also stop participating in the research at any time you choose. It is your choice, and all of your rights will still be respected.

Who to Contact

If you have any questions you may ask them now or later, even after the study has started. If you wish to ask questions later, you may contact any of the following:

Bernardo Abel Cedeñ o veloz(email: ba.cedeno.veloz@navarra.es)

This proposal has been reviewed and approved by Navarra Clinical Research Ethics Committee which is a committee whose task it is to make sure that research participants are protected from harm.

PART II: CERTIFICATE OF CONSENT

I have read the foregoing information, or it has been read to me. I have had the opportunity to ask questions about it and any questions that I have asked have been answered to my satisfaction. I consent voluntarily to participate as a participant in this research.

Name of Participant______________________________________

Signature of Participant __________________________________

Date ____________________________

Day/month/year

I have witnessed the accurate reading of the consent form to the potential participant, and had the opportunity to ask questions. I confirm that the individual has given consent freely.

Name of witness_________________________

Signature of witness ______________________

Date _________________________

Day/month/year

The researcher/person taking consent:

I have accurately read out the information sheet to the potential participant. I confirm that the participant was given an opportunity to ask questions about the study, and all the questions asked by the participant have been answered correctly and to the best of my ability. I confirm that the individual has not been coerced into giving consent, and the consent has been given freely and voluntarily.

A copy of this ICF has been provided to the participant.

Name of Researcher/person taking the consent________________________

Signature of Researcher /person taking the consent__________________________

Date ___________________________

Day/month/year
